# Data on Na,K-ATPase in primary cultures of renal proximal tubule cells treated with catecholamines

**DOI:** 10.1016/j.dib.2015.12.013

**Published:** 2015-12-25

**Authors:** Mary Taub, Facundo Cutuli

**Affiliations:** Biochemistry Department, University at Buffalo, 140 Farber Hall, 3435 Main Street, Buffalo, NY 14214, USA

**Keywords:** Catecholamines, Kidney, Proximal tubule, Na,K-ATPase, Chronic

## Abstract

This data article is concerned with chronic regulation of Na,K-ATPase by catecholamines. After a chronic treatment, inhibition of Na,K-ATPase activity was observed in cultures with dopamine, while a stimulation was observed in cultures treated with norepinephrine. Following a chronic incubation with guanabenz, an α adrenergic agonist, an increase in Na,K-ATPase α and β subunit mRNAs was observed. This data supports the research article entitled, “Renal proximal tubule Na, K-ATPase is controlled by CREB regulated transcriptional coactivators as well as salt inducible kinase 1” (Taub et al. 2015) [1].

**Specifications Table**

**Table:** 

Subject area	*Biology*
More specific subject area	*Renal transport regulation*
Type of data	*Figure*
How data was acquired	Real-Time PCR on a Biorad Cycler, ^86^Rubidium uptake studies
Data format	*Analyzed*
Experimental factors	Primary cultures of rabbit kidney proximal tubule cells treated with catecholamines and control
Experimental features	Rb^+^ uptake into intact cells was examined in triplicate, and standardized with respect to protein, to calculate *n*moles of Rb^+^ uptake per mg protein; in Real-Time PCR, Ct values of Na,K-ATPase and GAPDH mRNAs were obtained from quadruplicate determinations, and used to calculate the relative increase in Na,K-ATPase in catecholamine treated and control cells.
Data source location	All analyses and experiments were performed in Buffalo, New York, USA
Data accessibility	*Data is with this article*

**Value of the data**

• This data will have an impact on therapies using catecholamines for blood pressure regulation.
• The data can be compared with other studies of transcriptional regulation of the genes encoding for each of these subunits.

## 1 Data

The data shown in this report measures changes both in Na,K-ATPase activity and Na,K-ATPase mRNA levels following a chronic incubation of renal proximal tubule cells with catecholamines.

## 2 Experimental design, materials and methods

### 2.1 Rubidium uptake studies

**Fig. 1:**
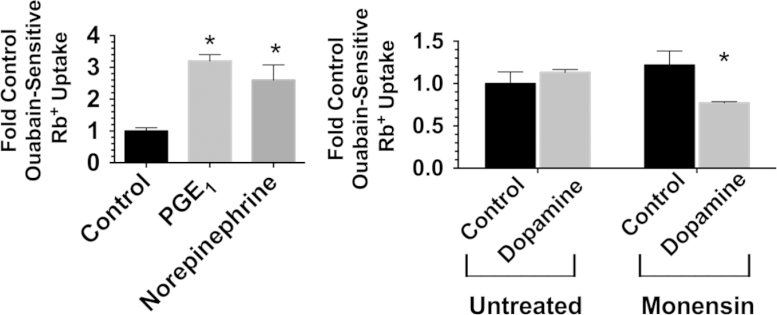
The effect of PGE_1_, norepinephrine and dopamine on transport. A. Primary RPT cells were incubated 30 min with either 280 nM PGE_1_, 1 µM norepinephrine or untreated (and +/−ouabain), followed by a 20 min uptake period with 1 mM ^86^Rb^+^. Uptake values are averages (+/−SEM) of ouabain-sensitive Rb^+^ uptake relative to the untreated control. The ouabain-sensitive component of Rb^+^ uptake was calculated by subtracting the Rb^+^ uptake observed in the presence of ouabain from total Rb^+^ uptake. The results were divided by the untreated control value. B. Primary RPT cells were incubated 30 min with either 10 µM dopamine +/−5 µM monensin, or untreated (+/−5 µM monensin). Uptake studies were conducted both in the presence and in the absence of 1 mM ouabain for each of the 4 conditions, followed by a 20 min uptake period with 1 mM ^86^Rb^+^. The ouabain-sensitive component of Rb^+^ uptake was determined as described in part A. *p<0.05 relative to untreated Control.

Primary cultures of rabbit kidney proximal tubule cells, were prepared as described previously [1,2]. Rabbits employed to obtain the primary cultures were used by procedures approved by the University at Buffalo Institutional Animal Care and Use Committee. The primary cultures were grown in a 50:50 mixture of Dulbecco׳s Modified Eagle׳s medium and Ham׳s F12 (DME/F12) supplemented with 5 µg/ml bovine insulin, 5 µg/ml human transferrin and 5×10^−8^ M hydrocortisone (i.e. Medium RK-1) [2]. ^86^Rb^+^ uptake studies were conducted, as described previously, in K^+^-free DME/F12 supplemented with insulin, transferrin and indomethacin [3], [4]. Agonists (PGE_1_, norepinephrine, Fig. 1A, and dopamine, Fig. 1B) were added after a 30 min pre-incubation in this supplemented, K^+^-free DME/F12 (+/−1 mM ouabain). Subsequently, agonists were added, followed by a 30 min incubation, the addition of ^86^Rb^+^ (1 mM), and a 20 min uptake period. Uptake values were corrected for zero time uptake, and standardized with respect to protein. The ouabain-sensitive component of Rb^+^ uptake was calculated, and divided by the value obtained for ouabain-sensitive Rb^+^ uptake with untreated controls (Fig. 1). Differences were considered significant if *p*<0.05 relative to the untreated control value.

### 2.2 Na,K-ATPase mRNA levels

**Fig. 2:**
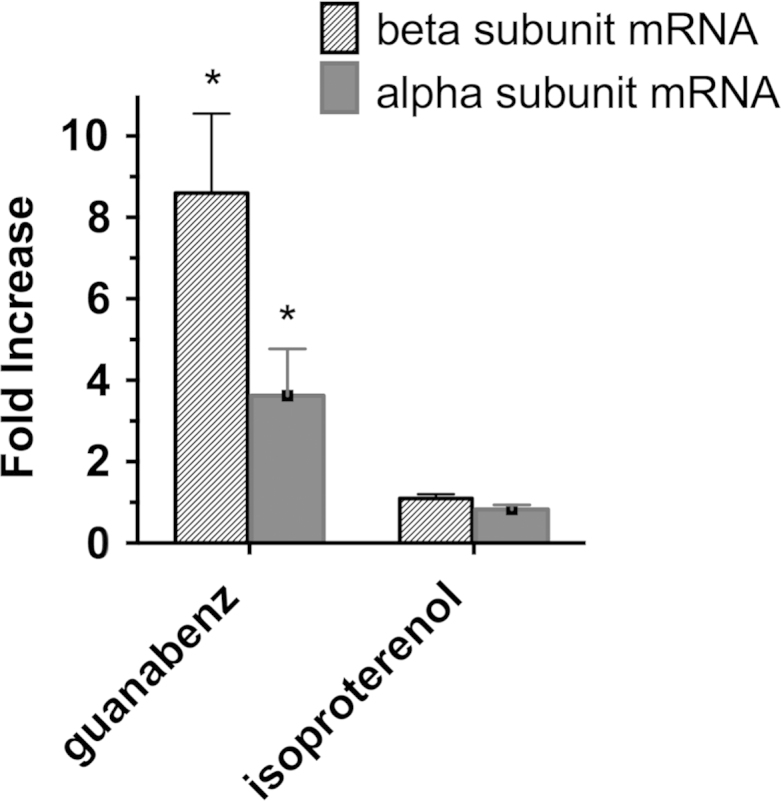
The effect of adrenergic agonists on Na, K-ATPase α and β subunit mRNAs. Primary RPT cells were incubated for 4 days in Medium RK-1 further supplemented with either 10 µM guanabenz, 10 µM isoproterenol, or untreated. After purifying RNA from the cultures, cDNA was synthesized, and the level of α and β mRNA was determined, relative to GAPDH mRNA. Values are averages (+/−SEM) of quadruplicate determinations, and were divided by the values obtained with untreated alpha and beta subunit mRNA controls. *p<0.05 relative to the untreated control alpha (or beta) subunit mRNA.

Primary RPT cells were incubated for 4 days with either guanabenz (an α adrenergic agonist), or isoproterenol (a β adrenergic agonist). Subsequently, RNA was purified, and cDNA was synthesized [4]. Specific cDNAs were amplified in a Bio-Rad iCycler. Ct values were calculated, and relative mRNA levels quantitated using GAPDH mRNA as an internal control. Primers designed using Primer-BLAST (NCBI), include primers for rabbit ATP1A1 (Assession number AF235024, ATP1A1_RABIT), TGACTCTCCTGCTCTGAAG, CACAATGGAAGCGAAGTTATC; rabbit ATP1B1 (Assession number AF204927, ATP1B1_RABIT), ACTGGCAAGCGAGATGAAG, ATGGTGAGGTTGGTGAACTG; and rabbit GAPDH (Assession number L23961, RABGLY3PHO), GCCCTCAATGACCACTTTGT, TCATGACAAGGTAGGGCTCC. Increases in the level of Na,K-ATPase α and β subunit mRNA were observed in the presence of guanabenz, and were considered to be significantly elevated relative to the untreated control if *p*<0.05 (Fig. 2).

## References

[1] Taub M., Garamella S., Kim D., Rajkhowa T., Cutuli F. (2015). Renal proximal tubule Na,K-ATPase is controlled by CREB regulated transcriptional coactivators as well as salt inducible kinase 1. Cell. Signal..

[2] Taub M. (2005). Primary kidney proximal tubule cells. Methods Mol. Biol..

[3] Taub M.L., Wang Y., Yang I.S., Fiorella P., Lee S.M. (1992). Regulation of the Na,K-ATPase activity of Madin–Darby canine kidney cells in defined medium by prostaglandin E1 and 8-bromocyclic AMP. J. Cell. Physiol..

[4] Herman M.B., Rajkhowa T., Cutuli F., Springate J.E., Taub M.L. (2010). Regulation of renal proximal tubule Na,K-ATPase by prostaglandins. Am. J. Physiol. Ren. Physiol..

