# Supplementary material for: Data on Na,K-ATPase in primary cultures of renal proximal tubule cells treated with catecholamines
Source: Data Brief. 2015 Dec 25;6:419–22. doi: 10.1016/j.dib.2015.12.013 (PMC4710796; doi:10.1016/j.dib.2015.12.013)
Supplement: Supplementary file 1 — Supplementary material [file mmc1.docx]

Conflict of Interest Statement

There are no known conflicts of interest associated with this publication and there has been no significant financial support for this work that could have influenced its outcome. The manuscript has been approved by named authors; there are no other persons who satisfied the criteria for authorship, and the order of authors listed in the manuscript has been approved. Due consideration has been given to the protection of intellectual property associated with this work and there are no impediments to publication, including the timing of publication, with respect to intellectual property. The regulations of our institutions concerning intellectual property have been followed. Any aspect of the work covered in this manuscript that has involved experimental animals has been conducted with the ethical approval of all relevant bodies and such approvals are acknowledged within the manuscript. The Corresponding Author a) is the sole contact for the Editorial process (including Editorial Manager and direct communications with the office), b) is responsible for communicating with the other authors about progress, submissions of revisions and final approval of proofs, and c) has provided a current, correct email address which is accessible by the Corresponding Author and which has been configured to accept email from the journal.
